# Effectiveness and postoperative wound infection of preperitoneal pelvic packing in patients with hemodynamic instability caused by pelvic fracture

**DOI:** 10.1371/journal.pone.0206991

**Published:** 2018-11-05

**Authors:** Hongjin Shim, Ji Young Jang, Ji Wan Kim, Hoon Ryu, Pil Young Jung, Seongyup Kim, Hye Youn Kwon, Kwang Min Kim, Hoejeong Chung, Keum Seok Bae

**Affiliations:** 1 Trauma Center, Wonju Severance Christian Hospital, Wonju, Korea; 2 Department of Surgery, Yonsei University Wonju College of Medicine, Wonju, Korea; 3 Department of Orthopedic Surgery, Asan Medical Center University of Ulsan, Seoul, Korea; 4 Department of Orthopedic Surgery, Yonsei University Wonju College of Medicine, Wonju, Korea; Virginia Commonwealth University, UNITED STATES

## Abstract

**Introduction:**

Despite the use of a multidisciplinary treatment approach, the mortality rate of hemodynamic instability due to severe pelvic fracture remains 40–60%. Several recent studies have shown that preperitoneal pelvic packing (PPP) was useful for achieving hemostasis in these patients in the acute phase. However, few studies have examined postoperative complications. The purpose of the present study was to evaluate clinical outcomes and wound infections of PPP in these patients.

**Materials and methods:**

We retrospectively reviewed the medical charts of 62 patients with hemorrhagic shock due to pelvic fracture between March 2011 and May 2017. Excluding four patients (two with other major hemorrhage sites and two who experienced cardiac arrest in the emergency room), the patients were divided into PPP (n = 30) and non-PPP (n = 28) groups according to PPP application. Clinical outcomes including early-stage mortality, transfusion amount, and surgical site infection (SSI) were compared between the two groups.

**Results:**

The overall mortality rate was 48.3% and the mean Injury Severity Score (ISS) was 39 ± 9. The 30 patients in the PPP group had a significantly lower hemorrhage-induced mortality rate than the 28 patients in the non-PPP group (16.7% vs 50%, p = 0.019), although both groups had similar patient characteristics (age, ISS, and initial serum lactate level). Independent factors associated with hemorrhage-induced mortality were PPP and the requirement of packed red blood cells for 4 h. In the PPP group, SSI occurred in 5 of 25 (20%) patients.

**Conclusions:**

PPP may be considered as a hemostatic modality for hemodynamic instability due to pelvic fracture because it reduces the hemorrhage-induced mortality rate. However, wound infections after the procedure should be considered.

## Introduction

The mortality rate of hemodynamically unstable patients due to pelvic fracture remains as high as 40–60% despite the use of various treatment modalities using a multi-disciplinary team approach [1–3]. Hemorrhage, the most common cause of death in these patients, mainly originates from injured veins and fractured pelvic bones [4, 5]. Surgical ligation or angioembolization (AE) of the internal iliac artery, pelvic binder, and pelvic external fixation (EF) have been commonly used to control pelvic fracture bleeding. Among them, AE and EF have been used in many institutions [6]. However, AE reportedly has limited usefulness in patients with hemodynamic instability because they must be transported to an interventional suite and a large time investment is required to prepare the intervention facility and radiologist. Ligation or AE of the bilateral internal iliac arteries sometimes causes serious complications such as perineal necrosis, bladder necrosis, gluteal muscle necrosis, sexual impotence [6]. Since the mid-2000s, studies on preperitoneal (retroperitoneal or extraperitoneal) pelvic packing (PPP) have reported that this simple and quick surgical technique was effective at controlling bleeding in unstable patients with pelvic fracture [7, 8]. Although some comparative studies (PPP vs AE or PPP vs non-PPP) were recently published [9, 10], most included small cohorts and were of descriptive nature [7, 11, 12]. Studies on the effectiveness of PPP or postoperative wound complications in these patients are very limited. Since May 2014, PPP has been performed for pelvic fracture patients with hemodynamic instability in the trauma center of Wonju Severance Christian Hospital. Therefore, the purposes of this study were to evaluate the effectiveness of PPP on hemorrhage-induced mortality and postoperative wound infection in hemodynamically unstable patients with pelvic fracture.

## Materials and methods

### Patient selection and data collection

This retrospective observational study was approved by the institutional review board of Wonju Severance Christian Hospital (IRB no. CR317114). The medical data of the pelvic fracture patients were selected from Wonju Severance Christian Hospital Pelvic Trauma Data Bank, which was collected prospectively by one trauma surgeon and developed as a part of the Korean Trauma Data Bank. Because the data were analyzed anonymously, informed consent was exempted. The inclusion criteria were: 1) hemodynamically unstable pelvic fracture, 2) age ≥ 20 years, and 3) agree to the collection and use of their medical information. Between March 2011 and May 2017, 531 patients were initially screened. Hemodynamic instability was defined as persistent hypotension (systolic blood pressure [SBP] < 90 mmHg) despite the loading of two units of packed red blood cells (RBCs) [13, 14]. The exclusion criteria were as follows: 1) another injury that caused a major hemorrhage, 2) patients with cardiac arrest at the time of arrival to the trauma bay, 3) isolated acetabular fracture, and 4) follow-up < 1 week. The patients’ characteristics that were obtained included age, sex, Injury Severity Score (ISS), initial hemoglobin, initial lactate, initial SBP, pelvic fracture type (Young-Burgess classification), open fracture, and combined injuries. Variables about patient management were also collected, including concurrent laparotomy or pelvic EF, pelvic binder, emergent pelvic angiography (PA), and requirement for packed red blood cells (RBC) for 4 hours and an additional 12 hours. Mortality, hemorrhage-induced mortality, duration of mechanical ventilation, and duration of intensive care unit (ICU) stay were collected as patient outcomes. Hemorrhage-induced mortality was defined as death caused by a persistent hypovolemic status requiring packed RBC transfusion after hemostatic procedures without other causes of death. Variables for postoperative wound infection were duration of surgical pad maintenance, timing, and name of the identified microorganisms.

### Patient management

The Wonju Severance Christian Hospital, has had a trauma team since 2010, and the trauma center building was inaugurated in January 2015. Before May 2014, for the treatment of pelvic fracture patients with hemodynamic instability in our institution, pelvic binder application, massive transfusion, and pelvic angiography (PA) were performed in cases of contrast extravasation on computed tomography. After May 2014, PPP was accepted for the same patients. Whether EF of the pelvic fracture was performed was decided by orthopedic surgeons of the trauma team and applied concurrently. We performed adjunctive PA when the bleeding persisted after PPP and EF. Resuscitative endovascular balloon occlusion of the aorta was not performed for any of the pelvic fracture patients during the study period. A pelvic binder was applied to reduce pelvic volume for all patients in the emergency room (ER) by trauma or orthopedic surgeons and removed just before the PPP or PA was started. In cases of EF application, the pelvic binders were not re-applied; rather, they were applied again just after PPP or procedure in patients without EF. After the patients became hemodynamically stable, the pelvic binders were removed and the trauma and orthopedic surgeons discussed the timing of definitive surgery for the pelvic ring injury. After the initial transfusion was started with two units of O-neg packed RBCs, cross-matched packed RBCs and fresh frozen plasma were administered at a 1:1 ratio according to our institution’s massive transfusion protocol.

### PPP techniques

PPP was performed by trauma surgeons who completed the Definitive Surgical Trauma Care course provided by the International Association for Trauma Surgery and Intensive Care [15]. After a 7–8-cm vertical skin incision was made starting at the symphysis pubis, resection of the anterior sheath of the rectus abdominis muscle and splitting of the muscle were performed. With medial migration of the peritoneum, blunt dissection was performed through the preperitoneal space in the posterolateral direction to the lateral border of the sacroiliac (SI) joint. Three surgical pads were packed firmly from the near side of the SI joint using ringed forceps. The same procedure was repeated on the contralateral side to facilitate tamponade and the skin was approximated with a continuous suture. After the coagulopathy, metabolic acidosis, and hypothermia were sufficiently corrected, according to the findings of the second look, the packed surgical pads were removed within 48 h [12]. Repacking was not performed due to the possibility of infectious complications.

### Evaluation of postoperative wound infection after PPP

Prophylactic antibiotics (second-generation cephalosporin) were administered preoperatively to patients who underwent PPP. Because surgical tape remained in the preperitoneal space after the PPP, the intravenous (IV) antibiotics were continued until the second look. Antibiotics were also added to every 10 units of packed RBC transfusions [16]. A surgical site infection (SSI) of PPP was diagnosed according to Centers for Disease Control and Prevention (CDC) guidelines [17]. The occurrence of SSI was described daily in the medical chart by trauma surgeons starting the day after the second look. When SSI was identified, a bacterial culture was obtained and IV antibiotics were started.

### Statistical analysis

Continuous variables are presented as mean ± standard deviation or median (range) and compared using Student’s t-test or the Mann-Whitney U test. Categorical variables are presented as frequency (percentage) and compared using the chi-square test or Fisher’s exact test. Logistic regression analysis was used to identify factors associated with hemorrhage-induced mortality, and the results are expressed as odds ratios (ORs) with 95% confidence intervals (CIs). P values < 0.05 were considered statistically significant, and all statistical analyses were performed using SPSS software (version 23.0; SPSS Inc., Chicago, IL, USA).

## Results

### Patient characteristics

**Fig 1:**
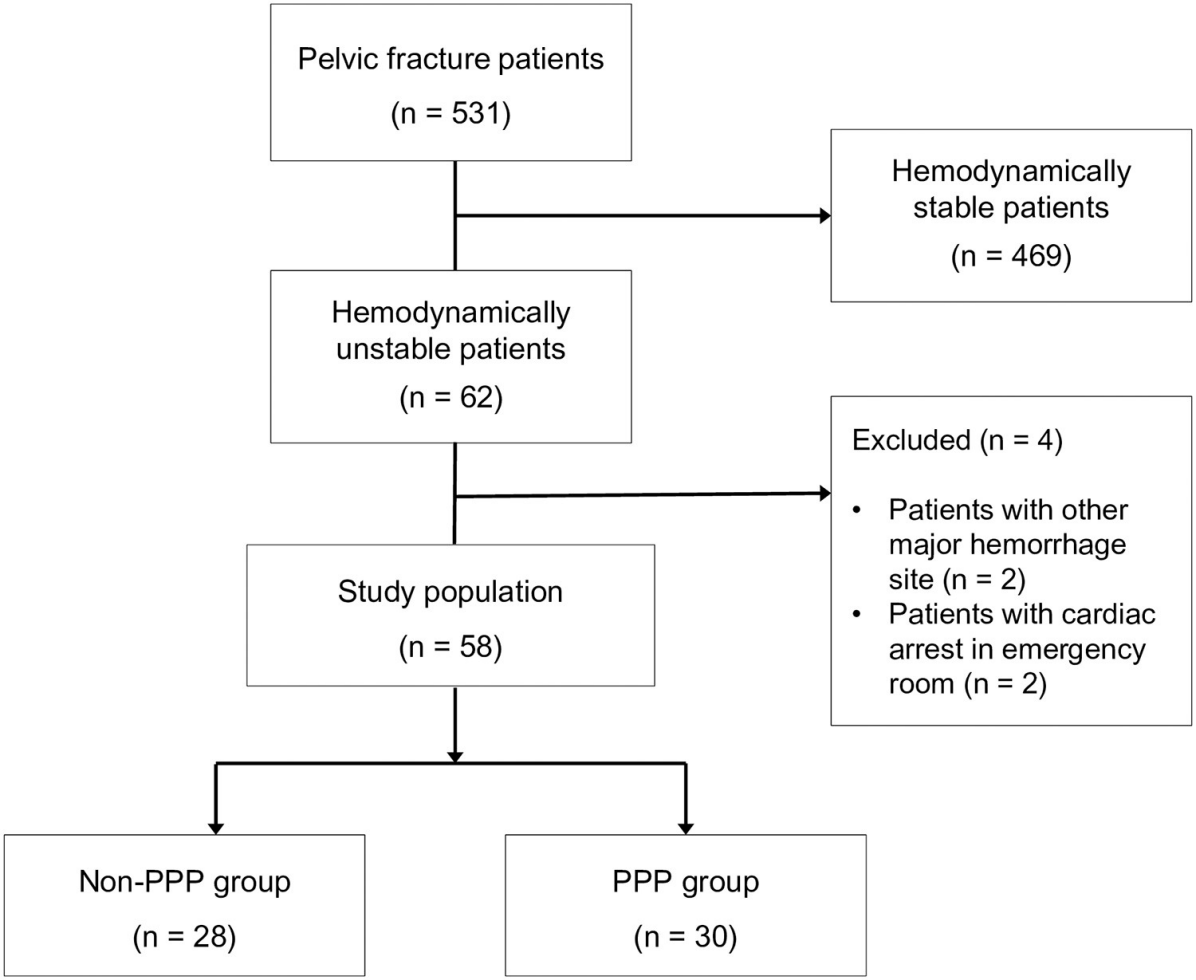
Study flow chart. PPP, preperitoneal pelvic packing.

Among the 62 pelvic fracture patients with hemodynamic instability, we excluded four patients: two with another injury causing major bleeding and two who experienced cardiac arrest upon ER arrival. Thus, 58 patients were enrolled in the present study (Fig 1). The mean age was 59.8 ± 18.9 years; 36 patients (62.1%) were male. The mean ISS was 39 ± 9, and 50 patients (86.2%) had associated injuries with an abbreviated ISS > 2. Thirty-three patients (56.9%) were transferred from other hospitals, and the mean serum lactate at the time of ER arrival was 5.20 ± 3.23 mmol/L. The most common injury mechanism was a pedestrian road traffic collision (RTC) (25/58; 43.1%), followed by a fall from a significant height (14/58; 24.1%), driver RTC (9/58; 15.5%), motorcycle RTC (4/58; 6.9%), passenger RTC (3/58; 5.2%), and a crush injury (3/58; 5.2%). According to the Young-Burgess classification of pelvic fracture type, 22 patients (37.9%) had lateral compression (LC) III, 18 (31.0%) had LC II, and 10 (17.2%) had vertical shearing(VS). Three patients (5.2%) had anteroposterior compression (APC) III type and LC I type, while two patients (3.4%) had APC II type. Pelvic EF was performed in 9 patients (15.5%), while pelvic AE was performed in 11 of 19 patients (32.8%) who underwent emergent PA. When the 28 patients in the non-PPP group and the 30 patients in the PPP group were compared, there was no significant difference in patient characteristics such as age, sex, ISS, pelvic fracture type, open fracture, the occurrence of a combined injury, and the collected initial physiologic parameters included initial hemoglobin level, initial lactate level, and initial SBP.

### Clinical outcomes of non-PPP and PPP groups

The overall mortality rate was 48.3%. Among the 28 dead patients, the most common cause of death was acute hemorrhage (19/58; 67.9%), followed by acute kidney injury (3/58; 10.7%), sepsis (2/58; 7.1%), acute respiratory distress syndrome (2/58; 7.1%), acute myocardial infarction (1/58; 3.6%), and delayed brain hemorrhage (1/58; 3.6%). Patients of the PPP group underwent pelvic EF more frequently than those of the non-PPP group (30% vs 0%, p = 0.002), but there was no significant difference in the frequency of emergent laparotomy (14.3% vs 23.3%, p = 0.508) and emergent PA (35.7% vs 30.0%, p = 0.517) between the two groups. The overall mortality rates were 57.1% in non-PPP group and 40% in the PPP group, but the difference was insignificant (p = 0.300). Five patients (16.7%) in the PPP group and 14 (50%) in the non-PPP group died of hemorrhage, and the intergroup difference was significant (p = 0.007) (Table 1).

**Table 1:** Non-PPP versus PPP groups.

	Non-PPP group (n = 28)	PPP group (n = 30)	P value
Age (years)	57.0 ± 22.8	62.5 ± 14.4	0.287
Sex (male)	16 (57.1)	20 (66.7)	0.455
Injury Severity Score	38.7 ± 9.2	38.4 ± 8.5	0.893
Transfer from other hospital	14 (50)	19 (63.3)	0.306
Initial hemoglobin (g/dL)	10.8 ± 3.0	9.9 ± 2.5	0.215
Initial lactate (mmol/L)	5.51 ± 3.56	4.90 ± 2.92	0.475
Initial SBP (mmHg)	71.8 ± 10.8	68.0 ± 9.8	0.172
Pelvic fracture type (Young & Burgess classification)			0.055*
APC type II	1 (3.6)	1 (3.3)	
APC type III	0	3 (10.0)	
LC type I	3 (10.7)	0	
LC type II	7 (25.0)	11 (36.7)	
LC type III	14 (50.0)	8 (26.7)	
VS type	3 (10.7)	7 (23.3)	
Open pelvic fracture	1 (3.6)	2 (6.7)	1.000*
Combined injury	22 (78.6)	28 (93.3)	0.138*
Concurrent laparotomy	4 (14.3)	7 (23.3)	0.508*
Pelvic external fixation	0	9 (30.0)	0.002*
Emergent pelvic angiography	10 (35.7)	9 (30.0)	0.517
Requirement of packed RBCs for 4 hours (units)	8.6 ± 5.2	12.1 ± 9.9	0.101
Requirement of packed RBCs for additional 12 hours (units)	2 (0–22) (19/28)	4 (0–24) (29/30)	0.669
Mortality	16 (57.1)	12 (40.0)	0.300
Mortality due to hemorrhage	14 (50.0)	5 (16.7)	0.007

APC, anterior posterior compression; LC, lateral compression; PPP, preperitoneal pelvic packing; RBCs, red blood cells; SBP, systolic blood pressure; VS, vertical shearing

*Result of Fisher’s exact test

### Independent factors associated with hemorrhage-induced mortality in pelvic fracture patients with hemodynamic instability

The patients with hemorrhagic mortality had a higher mean initial lactate level (6.59 ± 3.97 vs 4.52 ± 2.60 mmol/L; p = 0.049), a higher open fracture rate (15.8% vs 0%; p = 0.031), a higher requirement for packed RBCs for 4 h (14.8 ± 10.5 vs 8.2 ± 5.6; p = 0.003), were managed in trauma centers less frequently (26.3% vs 59.0%; p = 0.019), and underwent PPP less frequently (26.3% vs 64.1%; p = 0.007) than those without hemorrhagic mortality (Table 2). When logistic regression analyses were performed using these factors, the independent factors associated with hemorrhage-induced mortality were PPP (OR, 0.051; 95% CI, 0.008–0.318; p = 0.001) and requirement for packed RBCs for 4 h (OR, 1.251; 95% CI, 1.090–1.435; p = 0.001) (Table 3).

**Table 2:** Comparison of hemorrhagic mortality.

	No hemorrhagic mortality (n = 39)	Hemorrhagic mortality (n = 19)	P value
Age (years)	59.9 ± 15.4	59.7 ± 25.2	0.970
Sex (male)	22 (56.4)	14 (73.7)	0.203
Injury Severity Score	38.0 ± 7.3	39.7 ± 11.4	0.562
Initial hemoglobin (g/dL)	10.3 ± 2.3	10.5 ± 3.6	0.811
Initial lactate (mmol/L)	4.52 ± 2.60	6.59 ± 3.97	0.049
Initial SBP (mmHg)	70.9 ± 10.2	67.7 ± 10.7	0.273
Pelvic fracture type			0.204*
APC type II	1 (2.6)	1 (5.3)	
APC type III	2 (5.1)	1 (5.3)	
LC type I	1 (2.6)	2 (10.5)	
LC type II	15 (38.5)	3 (15.8)	
LC type III	12 (30.8)	10 (52.6)	
VS type	8 (20.5)	2 (10.5)	
Pelvic open fracture	0	3 (15.8)	0.031*
Combined injury	36 (92.3)	14 (73.7)	0.099*
Trauma center	23 (59.0)	5 (26.3)	0.019
PPP	25 (64.1)	5 (26.3)	0.007
Concurrent laparotomy	7 (17.9)	4 (21.1)	1.000*
Pelvic external fixation	8 (20.5)	1 (5.3)	0.247*
Emergent pelvic angiography	11 (28.2)	8 (42.1)	0.290
Requirement for packed RBCs for 4 h (units)	8.2 ± 5.6	14.8 ± 10.5	0.003

APC, anterior posterior compression; LC, lateral compression; PPP, preperitoneal pelvic packing; RBC, red blood cell; SBP, systolic blood pressure; VS, vertical shearing

*Results of Fisher’s exact test

**Table 3:** Independent factors associated with hemorrhagic mortality.

Variable	Risk factors for hemorrhagic mortality	
	Odds ratio (95% CI)	P value
Preperitoneal pelvic packing	0.051 (0.008–0.318)	0.001
Trauma center	0.478 (0.009–0.780)	0.476
Initial lactate	1.096 (0.843–1.424)	0.494
Requirement for packed RBCs for 4 h	1.251 (1.090–1.435)	0.001

CI, confidence interval; RBCs, red blood cells

### Clinical outcomes and wound infection patients who underwent PPP

**Fig 2:**
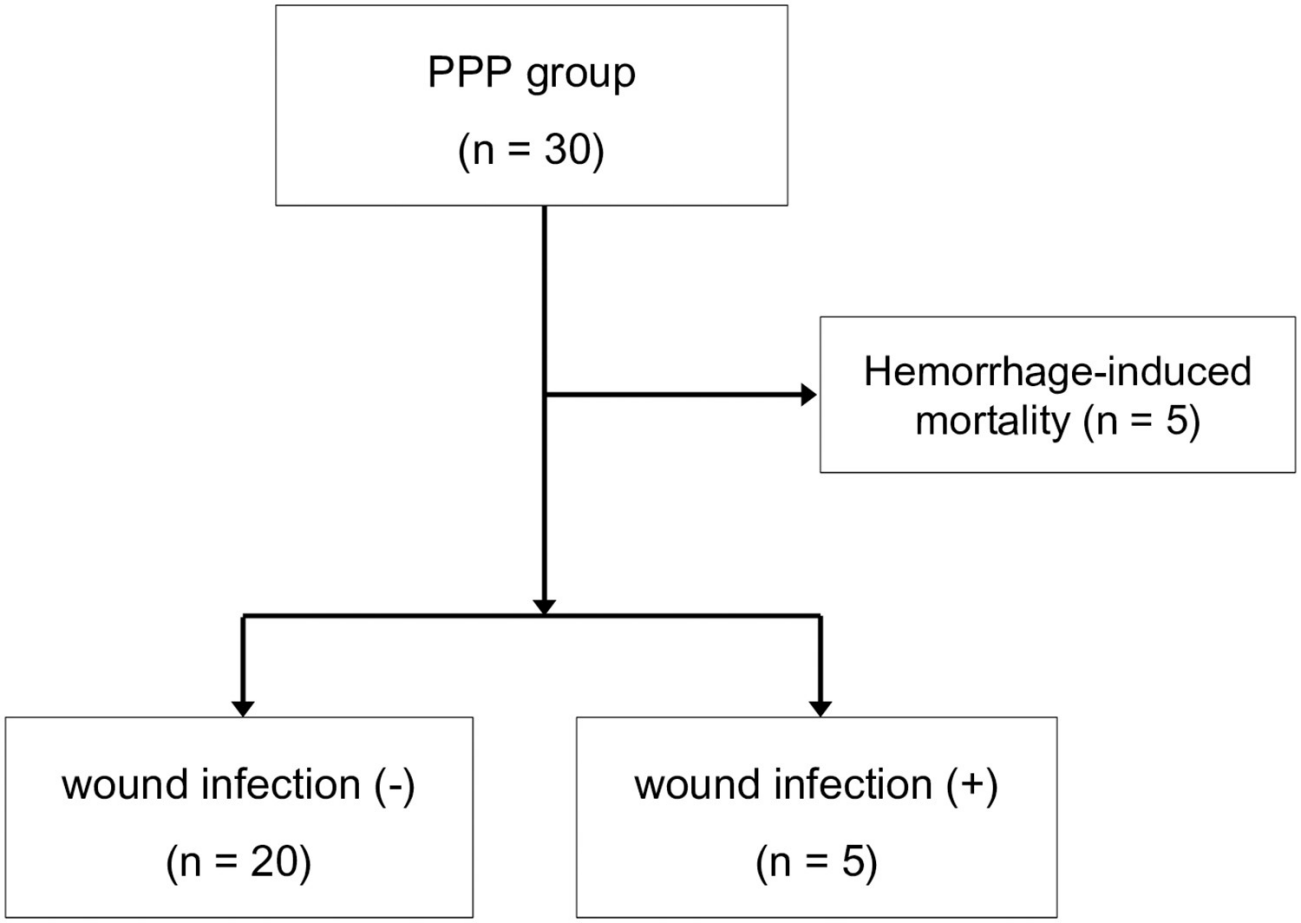
Postoperative wound infection in preperitoneal pelvic packing group. PPP, preperitoneal pelvic packing.

After 5 patients who died of exsanguination were excluded from the 30 patients who underwent PPP, clinical outcomes and wound complications of 25 patients were evaluated (Fig 2). The mean age was 60.7 ± 13.9 years; 15 patients (60%) were male. Three patients (12%) had diabetes mellitus and four (16%) were taking anticoagulants for their underlying diseases. The mean SBP was initially 68.2 ± 10.1 mmHg and the mean initial lactate level was 4.56 ± 2.62 mmol/L. Laparotomy in four patients (16%) and pelvic EF in eight (32%) were concurrently performed with the PPP. Packed surgical pads were removed after a mean 48.8 ± 20.8 h (median, 45 h [20–104 h] in patients without SSI vs 48 h [26–72 h] in patients with SSI; p = 0.891). Twelve patients (48.0%) underwent open reduction and internal fixation (ORIF) of the pelvis on the mean 8^th^ day of hospitalization. Among the eight patients who underwent emergent EF, ORIF was performed in four after EF removal, while the EF remained in the other four for 6–8 weeks. Four other patients were managed conservatively without EF or ORIF after PPP, and five patients were not able to undergo EF or ORIF because of mortality. A total of seven patients (28%) died, six (24%) of multi-organ dysfunction syndrome (acute kidney injury in three [12%], acute respiratory distress syndrome in two [8%], and SSI in one [4%]). The other died of a severe brain injury on the 30^th^ hospitalization day after successful hemorrhage control. A SSI was identified in five patients (20%): a deep incisional infection in three (12%), organ space infection in one (4%), and superficial incisional infection in one (4%). Of those five patients, a 79-year-old man (case no. 3; Table 4) with an APC type III fracture underwent a second operation for surgical pad removal and ORIF of the pelvic bone 56 h after the PPP, and the SSI was found on the second postoperative day after the second operation. *Enterococcus faecalis* was identified in the bacterial culture of his wound. He died of multiple organ failure on the third postoperative day after the second operation. A 35-year-old man with an APC type III pelvic fracture and a bladder injury underwent PPP and pelvic EF. A rectal injury was found on the third day after the second operation (surgical tape removal and primary repair of an extraperitoneal bladder rupture) despite a rectal examination upon ER arrival. After a temporary transverse colostomy, transanal repair of the rectum, and the administration of IV tazobactam/piperacillin, the SSI was controlled successfully and SI joint screw fixation was performed to stabilize the pelvic ring. *Escherichia coli* was identified in the bacterial culture of his incisional wound (case no. 5; Table 4).

**Table 4:** Case series of patients with surgical site infections.

No.	Age	ISS	DM	Pelvic binder	Pelvic EF	Time from PPP to second look (hours)	RBC transfusion (total)	Post-admission SSI identification timing	Organism	Mortality
1	51	38	X	O	O	57	8	5	MRSA	X
2	62	38	X	O	O	42	4	12	*Staphylococcus aureus*, *Enterobacter aerogenes*	X
3	79	38	X	X	O	72	8	6	*Enterococcus faecalis*, MDR AB (p)	O
4	70	35	O	X	X	48	11	8	*Enterococcus caseliflavus*	X
5	35	35	X	O	O	26	7		*Escherichia coli*	X

AB, *Acinetobacter baumannii*; DM, diabetes mellitus; EF, external fixation; MDR, multi-drug resistant; MRSA, methicillin-resistant *Staphylococcus aureus*; RBCs, red blood cells; SSI, surgical site infection

## Discussion

The present study showed that PPP was an independent preventable factor for hemorrhage-induced mortality and reduced hemorrhagic mortality by approximately 19 times compared to patients who did not undergo PPP (OR, 0.051; 95% CI, 0.008–0.318; p = 0.001). This result is similar to those of recently published studies [6, 11, 18]. Since PPP was first introduced by Pohlemann et al. in 1994, several studies have reported that it effectively controlled hemorrhage due to pelvic fracture. However, most of these studies described early experiences, descriptive cases, and retrospective designs [7, 8, 11]. Small comparative studies of PPP and PA showed that the mean time to hemostatic procedure in the PPP group was significantly shorter than that in the PA group and that there was no significant difference in the intergroup mortality rate [9, 19, 20]. Chiara et al. recently performed a propensity score analysis of 78 hemodynamically unstable patients whose major source of bleeding was the pelvis to adjust the baseline characteristics and severity of the two groups and reported that the early mortality rate was significantly reduced in the PPP than in the non-PPP group [10].

In our study, overall and hemorrhagic mortality rates were 40.0% and 16.7%, respectively, in patients who underwent PPP, and the overall mortality rate in particular was high compared to those of other previous studies, which were 33% [10], 21% [6], and 14% [9]. There are several reasons for this discrepancy. First, the mean age in the PPP group of our study was higher than those of the other studies (62.5 ± 14.4 vs 55.3, 44, and 43 years, respectively) [21]. We suggest that their vulnerability to volume overloading from massive transfusion and multiple organ failure due to sepsis was the cause of the high mortality rate after successful hemorrhage control in older patients. Second, because the Korean trauma system is currently under development and our trauma center is located in a mountainous area, prehospital management and inter-hospital transfers create many problems. Ours is the only certified trauma center in this area, and emergency centers of other hospitals in this area are less specialized for treating trauma patients. It seems that the high rate of transfer from other hospitals (63.3%) caused the relatively high mortality rate of our study. Third, the definition of hemodynamic instability due to pelvic fracture differed among studies, although “persistent hypotension despite the loading of 2 L of crystalloid and transfusion of 2 units of packed RBCs” has been mainly used [8, 10, 11, 19]. However, the Advanced Trauma Life Supports (ATLS) definition–SBP < 90 mmHg and heart rate > 120/min with evidence of skin vasoconstriction (cool, clammy, decreased capillary refill), altered level of consciousness, and/or shortness of breath[22]–was also used in several recent studies [14, 23]. Because patients who were enrolled with hemodynamic instability by the ATLS definition had less severe physiologic conditions, they had relatively lower mortality rates.

After acute hemorrhage was stopped in patients with hemodynamic instability due to pelvic fracture, trauma surgeons must make two important decisions about when to remove the packed surgical pads and perform definitive surgery for pelvic fracture. The timing of pelvic pack removal was recommended as within 24–48 hours after PPP in most previous studies; however, it was not based on evidence but experience [11, 14, 15]. In our data, there was no significant difference in the duration of the surgical pad remaining within the patients between those with or without an SSI. More evidence about this topic is needed in the future. The optimal time for ORIF of the pelvis is unclear and varies depended on patient’s condition. Generally, hemodynamically stable patients can be safely managed by early ORIF within 24 hours after the injury, while definitive fixation of the pelvic fracture should be postponed until after the 4^th^ day after the injury in patients with hemodynamic instability [14, 24]. Factors representing the patients’ general condition should also be considered, such as “no increasing infiltrate on chest radiography,” “balanced or negative fluid balance,” “PaO_2_/FiO_2_ > 250,” “platelet count > 95000/μL,” “maximal inspiratory airway pressure < 35 mmHg,” “white blood cell count < 12000/μL,” and “intracranial pressure < 15 cmH_2_O.” In our study, one old patient who underwent a second operation (surgical pad removal and ORIF of the pelvis) on the 3^rd^ postoperative day died of multiple organ failure. In cases in which pelvic fracture patients had obvious hemodynamic instability, trauma and orthopedic surgeons should closely discuss the timing of definitive pelvic fracture fixation.

Studies on post-PPP infection are very limited. Burlew et al. reported that the infection rate of the pelvic space after PPP was approximately 12% and were significantly higher in those patients undergoing repeat PPP (47%) than those after a single packing [6]. In a prospective randomized study, infections were identified in 3 (10%) of 29 PPP patients [9]. In our data, the SSI rate was 20% (5/25 patients who did not die of acute hemorrhage), classified by the CDC guideline as a deep incisional infection in three patients, organ space infection in one patient, and superficial incision infection in one patient. Four patients’ wounds were recovered by antibiotic treatment without surgical management. Although the wound infection rate was somewhat higher than those of the previous two studies, the result may be relatively acceptable considering the mean patient age in our study (60.7 ± 13.9 years).

The Denver Trauma Center in the US reported that protocolized care including PPP combined with EF resulted in excellent patient outcomes (21% of overall mortality rate) in 128 pelvic fracture patients with shock [6]. However, in our center, pelvic EF was performed in only nine patients (30%) among the patients undergoing PPP, and a pelvic binder was applied instead of EF and remained until the patient’s hemodynamic status was restored. In patients who underwent PPP with a pelvic binder, after the patient’s hemodynamic instability was corrected, ORIF of the pelvic fracture was performed by orthopedic surgeons [18]. In Korea, trauma teams mainly consist of emergency medicine physicians and trauma surgeons who are board certified in general or cardiothoracic surgery; however, orthopedic surgeons are scarce. Because orthopedic surgeons are often on on-call rather than in-hospital duty, emergency EF may not be performed immediately. In such situations, PPP is thought to play a pivotal role in the treatment of pelvic fractures since it is effective for achieving hemostasis and can afford time to prepare for other procedures such as PA. We expect that the trauma system will be well established in the future and that pelvic EF can be performed emergently with PPP.

This study has some limitations. First, there was no definitive protocol regarding the sequence of PPP and PA or whether to apply pelvic EF. Second, the number of patients is small and the study design was retrospective. Third, because most patients had combined injuries, it was difficult to accurately quantify the amount of bleeding caused by a pelvic fracture. Despite these limitations, this study showed that PPP is useful in hemostasis on multivariate analysis in pelvic fracture patients with hemodynamic instability and provides meaningful information about post-PPP infections. In the future, larger-scale prospective studies of this topic are needed.

In conclusion, PPP may be considered as a hemostatic method for hemodynamic instability due to pelvic fracture with other methods because it reduces the hemorrhage-induced mortality rate. However, post-procedural wound infections should be considered.
